# Role of the ECG in initial acute coronary syndrome triage: primary PCI regardless presence of ST elevation or of non-ST elevation

**DOI:** 10.1007/s12471-014-0598-9

**Published:** 2014-09-09

**Authors:** B. B. L. M. IJkema, J. J. R. M. Bonnier, D. Schoors, M. J. Schalij, C. A. Swenne

**Affiliations:** 1 Department of Cardiology, Leiden University Medical Center, PO Box 9600, 2300 RC Leiden, the Netherlands; 2 Department of Cardiology, Brussels University Hospital, Laarbeeklaan 101, B-1090 Brussels, Belgium

**Keywords:** Electrocardiogram, Triage, ST Elevation, Non-ST-Elevation, Complete occlusion, Incomplete occlusion

## Abstract

The major initial triaging decision in acute coronary syndrome (ACS) is whether or not percutaneous coronary intervention (PCI) is the primary treatment. Current guidelines recommend primary PCI in ST-elevation ACS (STEACS) and initial antithrombotic therapy in non-ST-elevation ACS (NSTEACS). This review probes the question whether this decision can indeed be based on the ECG. Genesis of STE/NSTE ECGs depends on the coronary anatomy, collateral circulation and site of the culprit lesion. Other causes than ischaemia may also result in ST-segment changes. It has been demonstrated that the area at risk cannot reliably be estimated by the magnitude of the ST change, that complete as well as incomplete occlusions can cause STE as well as NSTE ECGs, and that STE and NSTE patterns cannot differentiate between transmural and non-transmural ischaemia. Furthermore, unstable angina can occur with STE and NSTE ECGs. We conclude that the ECG can be used to assist in detecting ischaemia, but that electrocardiographic STE and NSTE patterns are not uniquely related to distinctly different pathophysiological mechanisms. Hence, in ACS, primary PCI might be considered regardless of the nature of the ST deviation, and it should be done with the shortest possible delay, because ‘time is muscle’.

## Introduction

There is ongoing debate concerning the difference in initial treatment in acute coronary syndrome (ACS) between patients presenting with and without ST-segment elevations in the ECG [1, 2]. The guidelines state that initial treatment of patients with ST-elevation ACS (STEACS, conventionally called STEMI: ST-elevation myocardial infarction) is percutaneous coronary intervention (PCI), possibly replaced by or preceded by thrombolysis if PCI is not or not timely available [3]. According to the guidelines, patients with non-ST-elevation ACS (NSTEACS, conventionally called NSTEMI: non-ST-elevation myocardial infarction) should initially receive antithrombotic medication, and when a patient does not have a high-risk profile, coronary angiography with or without PCI should only be performed as a rescue procedure after noninvasive therapy has proved to be ineffective [4, 5]. High-risk patients are defined as those with at least one of the following features: accelerating tempo of ischaemic symptoms in the preceding 48 h, prolonged ongoing (>20 min) rest pain, pulmonary oedema, new or worsening mitral regurgitation murmur, hypotension, bradycardia, tachycardia, >75 years of age, transient ST-segment changes larger than 0.5 mm, newly discovered bundle branch block or cardiac biomarker elevation [4].

Stratification of ACS patients into the STE and NSTE strata stems from the ‘thrombolysis era’ that preceded the current ‘PCI era’. In the early 1980s, when thrombolysis was still the predominant treatment modality, two seminal papers were published in which the efficacy of fibrinolysis was demonstrated in anterior myocardial infarction [6, 7]. Localisation of the ischaemia had been electrocardiographically assessed. In anterior and in ‘multiple location’ myocardial infarction, hospital mortality and 1-year mortality decreased, while in ‘ST depression’ infarction hospital mortality did not change significantly and 1-year mortality increased. Notwithstanding the incomparability of PCI and thrombolysis as reperfusion techniques, these strata were sustained into the PCI era, and caused the difference in preferential initial treatment, invasive/noninvasive, between the STEMI and NSTEMI groups. During the past decade, invasive rescue procedures in NSTEACS patients have shifted from ‘late’ towards ‘early’ PCI; however, within this patient group, PCI is termed ‘early’ if performed within 24 h [8]. Within such a time span, considerable necrosis may develop [9]. Also with NSTEACS, ‘time is muscle’, and the sooner reperfusion is accomplished, the better. Is initial waiting for success of antithrombotic treatment in NSTEACS the optimal therapy for these patients, or should PCI be the initial treatment in all ACS patients with persisting signs of ischaemia at rest and no improvement after vasodilator therapy, irrespective of the manifestation of the ECG?

In this overview, we address a number of questions related to the role of the ECG in the first hours of ACS, including the prehospital and the initial hospital phase. Typically, myocardial salvage possibilities are largest during the ‘golden hours’ after coronary occlusion. Salvage possibilities decrease quickly with time: Hedström and colleagues [9] demonstrated that, in man, 3 h after onset of chest pain already about 25 % of the area at risk has become necrotic.

This paper focuses on this hyperacute phase of ACS. Given appropriate logistic conditions, this is the time frame within which reperfusion of the occluded coronary segment can be attained in practice. The following questions are addressed: can the ECG, and more specifically the presence or absence of ST elevation or non-ST elevation, contribute to:

- Diagnosis of ACS;
- Differentiation between unstable angina and ongoing ischaemia/infarction;
- Assessment of the size of the area at risk;
- Assessment of the transmurality (transmural vs. non-transmural) of the ischaemia;
- Assessment of the degree of occlusion of the culprit coronary artery lesion (complete/incomplete occlusion).

The available information at first medical contact is limited to patient history, clinical symptoms, ECG and the response to vasodilator administration. Within the golden hours, it is often possible to get the results of initial biomarker analysis and echography. Hence these diagnostic options will also be addressed briefly.

- ** ACS diagnosis **

ACS diagnosis in the hyperacute phase is mainly based on patient history, symptoms and physical examination. The ECG taken upon initial medical contact can be used to assist in the decision whether the presenting symptoms are of cardiac origin and are an expression of ACS. Typical ST-segment changes in the ECG become manifest at a very early stage, actually within 1 min after ischaemia onset, and even preceding the onset of chest pain [10]. Despite the fact that the sensitivity of the ECG is not high, it remains an important tool to assist in a rapid establishment of the working diagnosis of ACS [11, 12].

The ECG can be used to categorise ACS into STEACS or NSTEACS. However, there are causes of STE [13–15] and of NSTE [16] other than ischaemia / ACS. Preexisting ST-segment abnormalities and conduction disturbances may hamper ischaemia detection in the ECG [17, 18]. On the other hand, absence of ECG abnormalities does not exclude ACS. Therefore, patient history remains of major importance [15].

- **Differentiation between unstable angina and emerging infarction**

According to the current guidelines, ACS is classified into either STEACS or NSTEACS / unstable angina (UA) [3–5]. UA is defined as transient ischaemia at rest with no evidence of necrosis (no positive cardiac biomarkers or Q waves). NSTEACS is diagnosed when also a rise in cardiac biomarkers is detected (necrosis is detectable) [5, 19].

In our view, it is not correct to associate UA uniquely with NSTE ECGs. Also ischaemia with STE can fade away, and this should be termed unstable angina as well. In general, unstable angina can occur as a consequence of the fact that an initially occluded coronary artery spontaneously opens in the course of ACS [20–22]. Kovacs and Yamamoto [21] revealed that a complete coronary occlusion was found in 90–95 % during angiography within 4 h after onset and this fell to 50–63 % at 12–24 h after onset. The restoration of coronary flow can be explained by two mechanisms, namely resolution of coronary artery vasospasm and/or the dissolution of thrombocyte aggregation [19, 22].

Differentiation between unstable angina and ongoing infarction can only be done *post hoc*; at the earliest 1–3 h after onset of ischaemia as this is the minimal time span needed to demonstrate a detectable change in the cardiac biomarkers [23]. Therefore, the characterisation of angina as ‘unstable’ cannot play a role in the triaging decisions in the golden hours: appropriate treatment would be unnecessarily delayed by waiting for eventual spontaneous resolution of the occlusion.

- **Assessment of the area at risk**

The magnitude of the ST changes (∆ST) is only weakly related with the area at risk. TerHaar et al. [17] analysed ECGs recorded during elective PCI procedures with long (till 5 min) balloon occlusion times, data from a study done in the pre-stent era. Actually, this setting can be regarded as a human model for the initial minutes of acute coronary syndrome. The correlation of the ST changes with sestamibi-assessed area-at-risk measurements was low (r = 0.62). This is likely to be explained by the cancellation effect, due to which small changes in the ST segment can occur in the presence of relatively large areas at risk. A large, curved ischaemic area can thus cause a small ST-amplitude change on the ECG. In conclusion, the size of the ST change does not give a reasonably accurate estimation of the size of the area at risk. Birnbaum et al. [24] mention, as an example, the proximal occlusion of a dominant left circumflex artery before the first obtuse marginal branch. This will cause transmural ischaemia of both the basal, mid-anterolateral, and inferior regions and may result in only minimal ST deviations in the limb leads.

- **Assessment of the transmurality of the ischaemia**

It is generally assumed that STEACS is associated with transmural ischaemia whereas NSTEACS is mostly associated with non-transmural subendocardial ischaemia [24]. Sarafoff and colleagues [12] quantified size and transmural extent of the infarcted area by contrast-enhanced cardiac magnetic resonance imaging in 220 patients and compared this with their admission ECG (57 % had STE ECGs and 43 % had NSTE ECGs). The infarction was transmural in 63 % of the STEACS patients and in 27 % of the NSTEACS patients. Because necrotic tissue (infarcted area) was measured in this study, the ischaemic area / area at risk in the hyperacute phase of ACS has extended further. Hence, the assumption that STE/NSTE ECGs represent transmural/non-transmural ischaemia is therefore incorrect, the difference is more gradual. NSTEACS may be associated with a smaller percentage of transmural ischaemia than STEACS; however, with 27 % of the patients having transmural ischaemia this is still a significant number of patients.

- **Assessment of the degree of occlusion**

Some publications suggest that STE and NSTE ECGs are caused by complete, prolonged and by incomplete, temporal occlusions of a coronary artery segment, respectively [25–27]. The current guidelines [4] state that NSTE ECGs can occur with complete and with incomplete occlusion. In contrast, the current STEACS guidelines do not make this distinction [3]. Several studies have shown that complete and incomplete occlusion can produce STE as well as NSTE ECGs. Studying a series of 300 ACS patients with a completely occluded culprit artery and treated by primary PCI, it was found that 28.7 % of these patients had an NSTE ECG [28]. Studying NSTEACS patients, Wang et al. [29] and Bahrmann et al. [30] report occluded (Thrombolysis in Myocardial Infarction flow grade 0 to 1) culprit arteries in 27 and 29 % of the patients, respectively. Occlusions were more often found in coronary arteries supplying the inferolateral or posterolateral myocardium; patients with an occluded culprit artery had larger infarcts and a higher 6-month mortality [29, 30]. Knot et al. [26] found that the infarct-related artery was occluded in 66 % of the STEACS patients and in 35 % of NSTEACS patients with an ST-depression ECG (ST depression was defined as ‘new horizontal or down-sloping ST depression ≥ 0.05 mV in two contiguous leads or transient ST-segment elevations’). Koyama et al. [31] found occluded culprit arteries in 60 % of the STEACS patients and in 51 % of the NSTEACS patients. The prevalence of coronary flow limitation in NSTEACS patients was almost as high as in STEACS patients. In their study, Koyama and colleagues applied an acute PCI procedure treatment strategy to ‘all patients with non-ST elevation acute coronary syndromes if symptoms and/or electrocardiographic abnormalities did not respond to anti-ischaemic treatment within 20 min and acute myocardial infarction (AMI) was suspected by clinicians.’ Using this treatment strategy, they achieved an average treatment delay, calculated from onset of symptoms, of 203 and 292 min in the STE and NSTE patients, respectively.

Ter Haar and colleagues [17] analysed a database of ECGs taken during long balloon occlusions in elective percutaneous transluminal coronary angioplasty procedures. Occlusion sites were the left main coronary artery (2 %), left anterior descending (30 %), right coronary artery (49 %), and left circumflex (19 %). After 3 min of complete occlusion, 55 % of the patients had an STE ECG and 45 % had an NSTE ECG. This demonstrates directly how occlusions at various locations in the coronary arterial system may cause STE as well as NSTE ECGs.

In conclusion, both complete and incomplete coronary artery occlusions can cause both STE and NSTE ECGs and, hence, STE and NSTE ECGs do not discriminate complete and incomplete coronary artery occlusions.

- **Early biomarker analysis**

Guidelines state that the diagnosis of myocardial infarction is based on a rise in cardiac biomarker concentrations, demonstrating necrosis [3, 4]. Van der Laarse et al.(23) describe that determining cardiac troponin I/T in the hyperacute phase of ACS is not yet sensitive enough. Sebbane et al. [32] and Afzali et al. [33] revealed that the detection of the combination of troponin and copeptin could be used to rule out NSTEACS and could help identify which patients were suitable for discharge (sensitivity increased from 76 % (troponin alone) to 96 % (combination of troponin and copeptin)). Charpentier et al. [34] stated that the measurement of the combination of troponin and copeptin improved the diagnostic accuracy for NSTEACS patients, but the sensitivity (90.4 %) was too low to rule out NSTEACS.

Irrespective of the diagnostic modality, it takes at least 1–3 h after the onset of symptoms to detect such a rise and, actually, waiting this long before the decision to perform PCI is taken implies that already substantial damage to the myocardium may occur [23].

Concluding, the early measurement of cardiac biomarkers is not sensitive enough to diagnose emerging infarction in the initial stage of ischaemia, and waiting for a demonstrable rise in biomarkers creates an unacceptable time delay.

- **Echocardiography**

In the ischaemic cascade, mechanical dysfunction precedes electrical dysfunction and angina [10]. Echocardiography is generally accessible in the emergency department and can help to diagnose ACS; this is especially useful when the ECG is inconclusive [35]. In an early study in the Netherlands, Peels and colleagues reported, with two-dimensional echocardiography, 92 % sensitivity, 53 % specificity and 94 % negative predictive accuracy in patients admitted to the emergency department with acute chest pain and a non-diagnostic ECG [36].

## Discussion

Angioplasty to restore coronary blood flow was first performed in an elective procedure by Gruentzig in 1977 [37]. Soon thereafter, the first reports appeared describing the results of angioplasty in acute myocardial infarction patients [38, 39]. In the early 1980s thrombolysis was still the predominant treatment modality, and in 1986 and 1987 two seminal papers appeared in which the efficacy of fibrinolysis was demonstrated in anterior myocardial infarction [6, 7]. Localisation of the ischaemia had been electrocardiographically assessed. In anterior myocardial infarction hospital mortality decreased from 18.4 % (saline infusion, control) to 14.5 % (streptokinase infusion); 1-year mortality decreased from 26.0 to 22.1 %. In ‘multiple location’ infarction, hospital mortality decreased from 13.9 to 9.0 % and 1-year mortality decreased from 17.9 to 14.4 %. In ‘ST depression’ infarction, hospital mortality did not change significantly and 1-year mortality increased from 24.2 to 34.0 %. From then on, the leading thought emerged that reperfusion attempts were beneficial in anterior myocardial infarction and were detrimental in ‘ST depression’ myocardial infarction, where antithrombotic treatment became the predominating initial therapy. Notwithstanding the incomparability of PCI and thrombolysis as reperfusion techniques, this concept has sustained into the PCI era, and this explains why there is little evidence about the efficacy of primary PCI in NSTEACS.

Whether a patient will develop an STE or an NSTE ECG during ischaemia depends on the location of the culprit lesion and on the existence and extent of collaterals. Menon and colleagues [40] and Wong and colleagues [41] demonstrated that occlusion of the circumflex coronary artery occurs more often in NSTE ECGs (42.5 %) than in STE ECGs (11.2 %). The development of collaterals determines the extent of ischaemia and this can have either unexpected severe or irrelevant effects. Bahrmann et al. [30] showed that well-developed collaterals can limit the extent of myocardial damage and can improve the clinical outcome of the patient [42]. However, the existence of collaterals can also determine the extent of ischaemia in a negative manner in cases where the occlusion reduces the blood flow to the area supplied by the collaterals [43]. These considerations underline that there is no simple way to separate STEACS and NSTEACS patients; the underlying and sometimes individually very different anatomy of the coronary artery tree and the collateral circulation can create overlapping electrocardiographic manifestations.

In ACS, the prevalence of NSTEACS is higher (60 %) than STEACS (40 %).(44) The average characteristics of STEACS and NSTEACS patients differ. NSTEACS patients are in general 4 years older, more often female and more often have multivessel disease (41.1 % vs. 29.9 %); STEACS patients are more often haemodynamically unstable at presentation [44, 45].

The most recent NSTEACS guidelines [46] state that immediate PCI in NSTEACS patients has no advantages but also no disadvantages for the patients. The evidence for this statement is, however, limited: it is based on a single investigation, in which the outcome was based on the peak troponin level [47]. Some reports have been published about the experience with primary PCI in NSTEACS patients, and compared the results with those in STEACS patients [1, 26, 29, 31]. (Knot et al. [26] restricted the NSTEACS group to patients with ST depression). The results suggest that primary PCI in NSTEACS patients is recommendable. Katritsis et al. [48] demonstrated that PCI soon after admission of NSTEACS patients significantly reduced the risk for recurrent ischaemia (relative risk 0.59) and the duration of hospital stay (by 28 %). Furthermore, there was a decrease in major bleeding events (relative risk 0.78) and less death, MI or stroke (relative risk 0.91). Birnbaum and colleagues [11, 49] recently compiled a number of NSTE patterns (patterns associated with ongoing ischaemia or with reperfusion), which would require revascularisation by PCI because of the associated high risk.

Birnbaum et al. [24] provide an overview of the many similarities between the pathology underlying STE and NSTE ECGs and explain the physical concepts underlying the genesis of the ECG and why ischaemia at certain locations manifests with STE and at other locations manifests as NSTE. This view has partly been adopted by current clinical practice, because ‘true’ lateral (formerly termed posterior) ischaemia that manifests as ST depression in leads V1 through V3 is now called and treated as STEACS.

The size of the infarct is important for the prognosis after the initial treatment of ACS [9]. Coronary artery disease is the most common cause of heart failure (HF, in 50–70 % of the cases) [50]. Also smaller infarctions can lead to heart failure [3, 51]. Goel et al. [50] showed that every 1-h delay in performing PCI is associated with 4–12 % increased risk of new-onset heart failure and a 4 % relative increase in the risk of incident heart failure during follow-up. This research underscores the urgency to decide about the initial treatment strategy in NSTEACS.

In evidence-based medicine, decisions about the optimal treatment of patients are taken on the basis of their risk stratification. In ACS, the hazard of maintaining the STEACS and NSTEACS strata originating from the pre-PCI era is that NSTEACS patients have delayed access to PCI. This can lead to unnecessarily large necrotic areas, with negative consequences for the prognosis of these patients.

If, in addition to STEACS patients, also NSTEACS patients were to be candidates for primary PCI, this would grossly increase the amount of emergency catheterisations. It is difficult to predict the economic consequences of such a dramatic change in treatment strategy. On one hand, it is known that PCI treatment is expensive, especially in NSTE patients [52]. On the other hand, rescue PCI in NSTE patients is often performed late, up to 24 h after start of the symptoms, sometimes after transportation from a non-PCI equipped hospital to a hospital with PCI facilities. These conditions together may partly explain the increased costs. Swift primary PCI in these patients might make the costs more comparable with those of the STE PCI patients and might considerably change the further course of the emerging infarction scar and the associated cardiac function, and have influences on both the current hospital stay and on the long-term prognosis of this patient. If swift emergency PCI is the best way to treat these patients, the associated costs would be more likely to decrease than increase.

Actually, adopting primary PCI as the treatment of choice in STEACS as well as in NSTEACS creates a new stratum of patients in whom the optimal treatment strategies are still to be established. An initial important choice would be the selection of the type of stent to be used in the primary PCI procedure. Comparisons made between drug-eluting stents in STE and NSTE patients, such as the everolimus-sirolimus comparison study by Velders et al., [53] have to be repeated for this new stratum. Also, the medication regimen in this patient group should be reconsidered.

With the vanishing STE / NSTE classification, the direction of the ischaemia vector in the ECG is no longer relevant. Instead, measuring the ischaemia vector size, or a surrogate measure thereof, as proposed by Meissner and colleagues [54], would be more appropriate. For sufficient sensitivity the detection threshold for ischaemia in the ST segment should be lower than the current values of 0.1 mV; possibly this should go down to 0.05 or even 0.025 mV [18]. Such a low detection threshold is difficult to apply in the many patients who have preexisting non-zero ST segments or conduction defects. Also, it would fail, as do the current criteria, in patients with preexisting conduction disturbances / wide QRS complexes. This intrinsic problem in ischaemia detection in the ECG can be addressed by serial ECG analysis (comparison of the acute ECG with a previous ECG of the same patient that serves as a baseline ECG) [17, 18, 55].

**Fig. 1:**
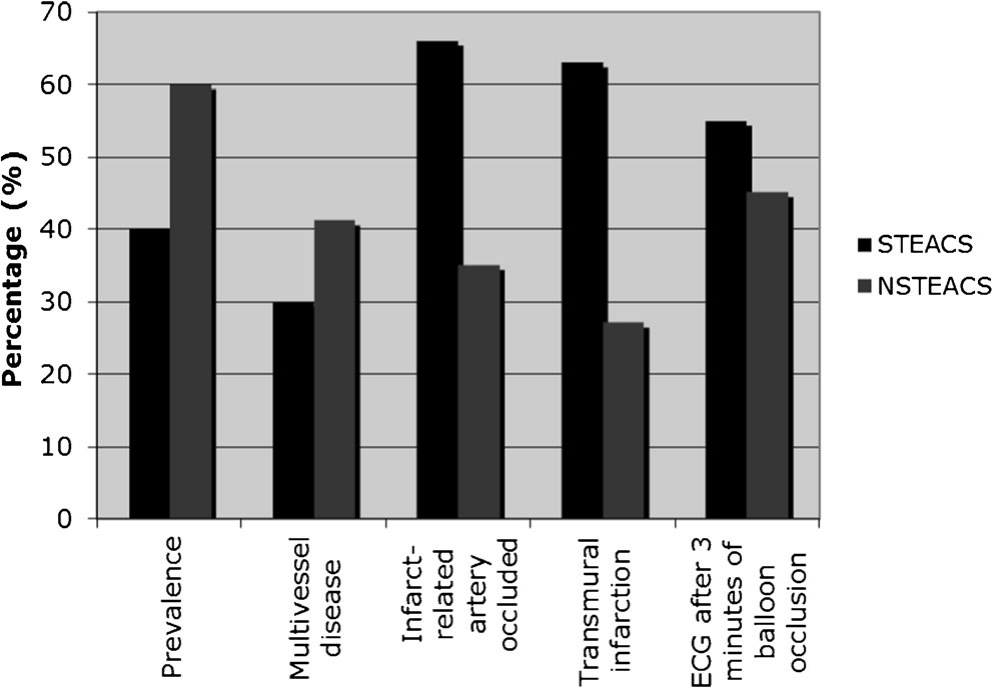
STE and NSTE acute coronary syndrome (STEACS and NSTEACS) are syndromes without a sharp contrast: properties overlap and differences are gradual. *Prevalence*: NSTEACS occurs slightly more often than STEACS [44]; *multivessel disease*: NSTEACS patients have slightly more often multivessel disease [44]; *infarct*-*related artery occluded*: this occurs more often in STEACS patients but also in a considerable percentage of NSTEACS patients [26, 28–31]; *transmural infarction*: this occurs more often in STEACS patients but also in a considerable percentage of NSTEACS patients [12]; *ECG after 3 min of balloon occlusion*: in elective PCI, only a slight majority of ECGs after 3 min of complete occlusion shows ST elevation [17]

In conclusion, and summarized in Fig. 1

, electrocardiographic STE and NSTE patterns are not uniquely related to distinctly different pathophysiological mechanisms; in our view, prompt PCI should be considered in all ACS patients, irrespective of whether ischaemia manifests as STE or as NSTE.
